# Supplementary material for: Mapping climate adaptation corridors for biodiversity—A regional-scale case study in Central America
Source: PLoS One. 2024 May 31;19(5):e0304756. doi: 10.1371/journal.pone.0304756 (PMC11142673; doi:10.1371/journal.pone.0304756)
Supplement: S1 Appendix — Supporting document containing supplementary figures and tables. (DOCX) [file pone.0304756.s001.docx]

**Supporting Information**


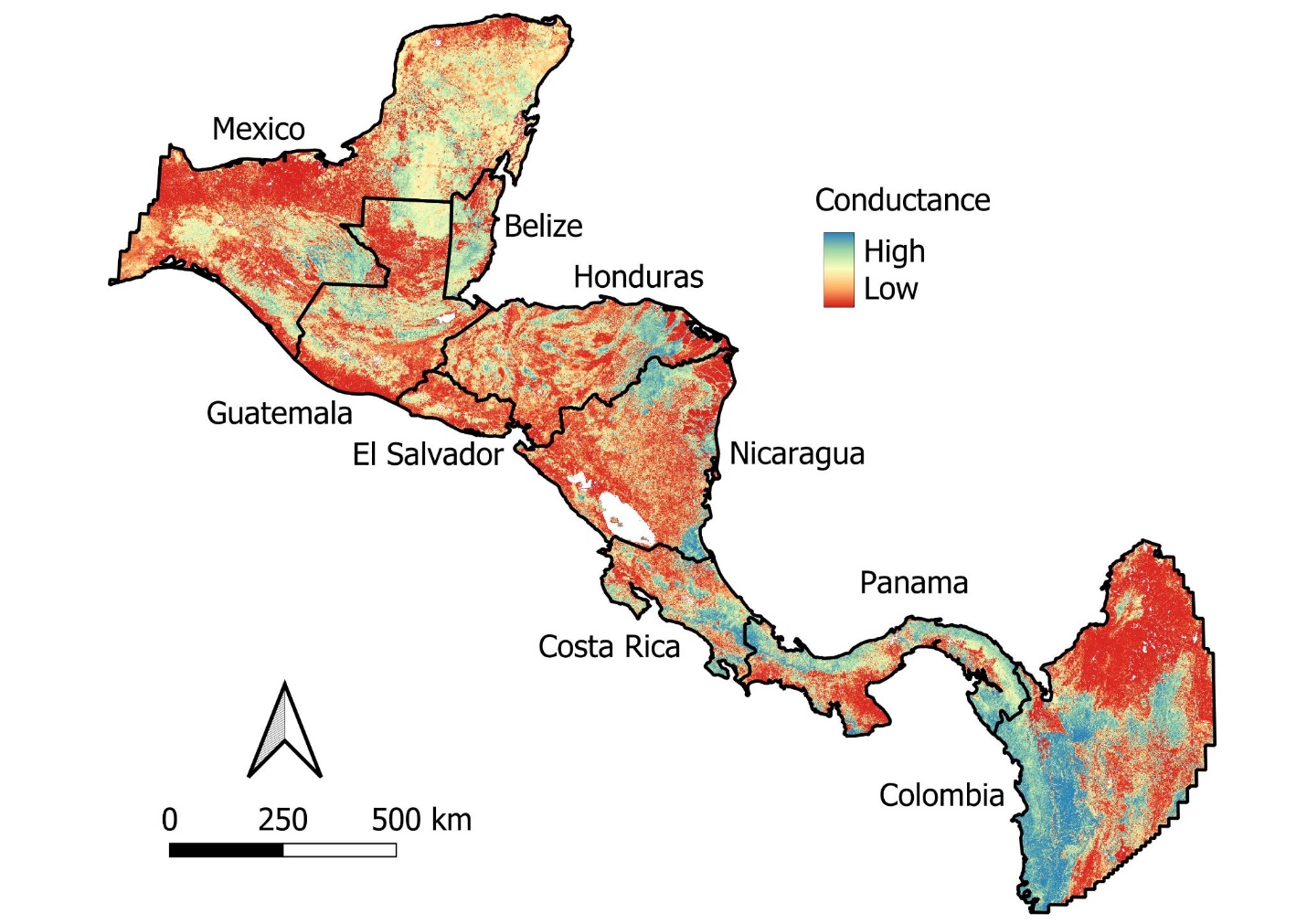


**S1 Fig. Conductance surface (100 m resolution) used for least cost path modeling.** Country boundaries are public domain and were obtained from Natural Earth (50m-admin-0-countries-2) before clipping to our study area (<https://www.naturalearthdata.com/>).


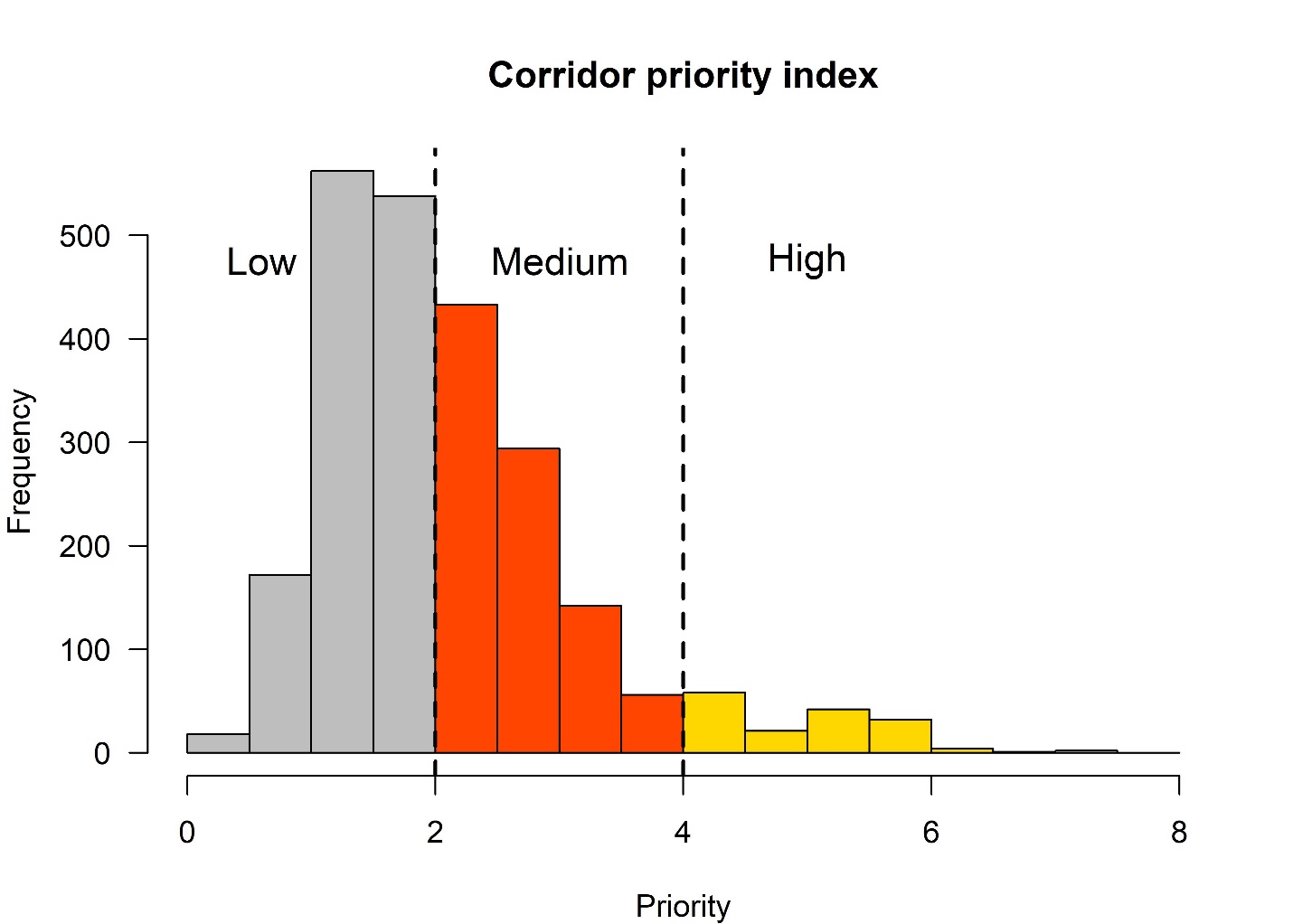


**S2 Fig. Distribution of climate adaptation corridor priority index values by level.** Low: n=1290, Medium: n=925, High: n=160.


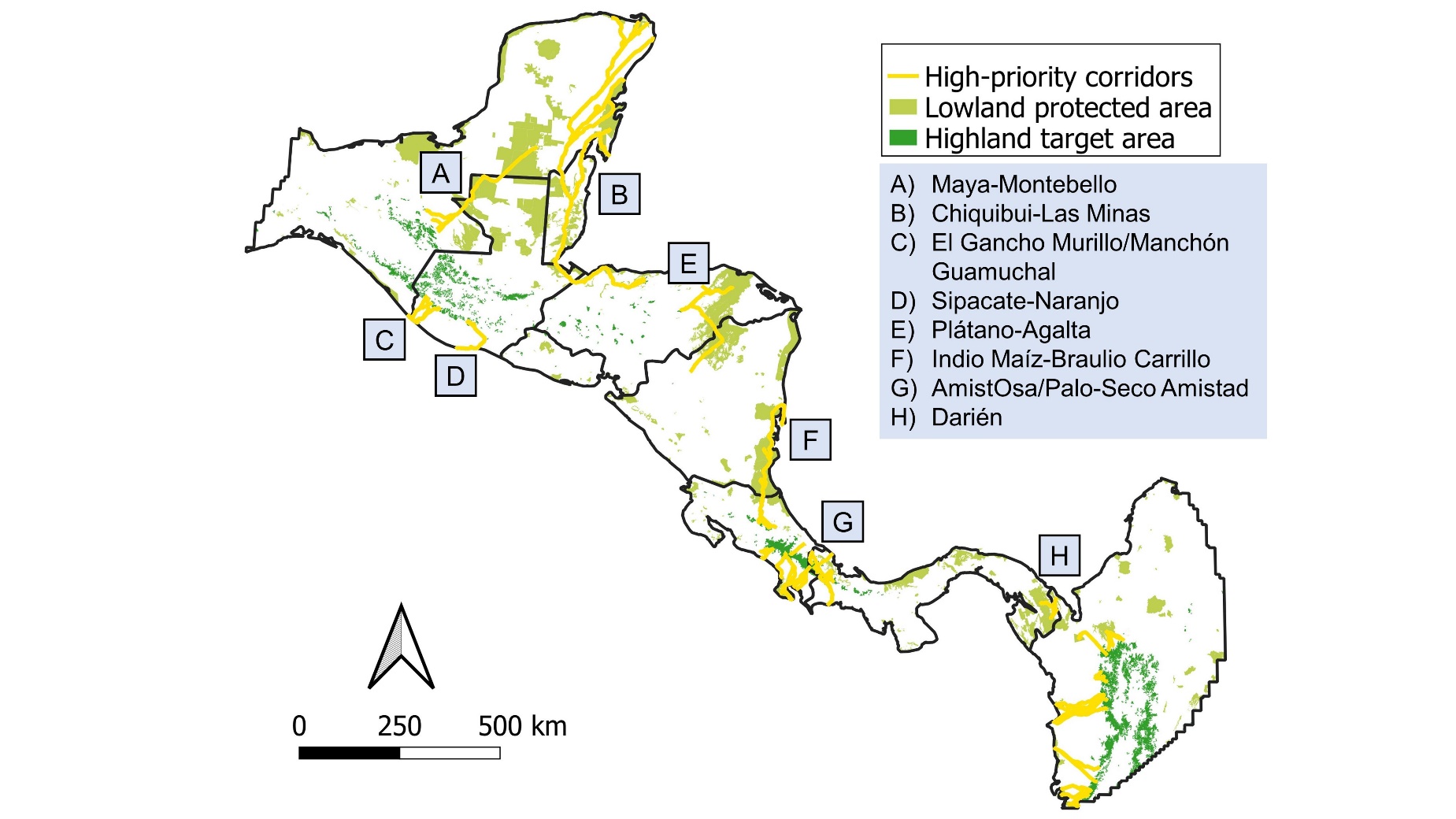


**S3 Fig. High-priority landscapes for future, finer-scale research, conservation, restoration, or investment activities based on relatively high densities of high-priority climate adaptation corridors in Central America.** We omitted areas fully in Colombia because they were outside our focal study area. Country boundaries are public domain and were obtained from Natural Earth (50m-admin-0-countries-2) before clipping to our study area (<https://www.naturalearthdata.com/>). Lowland protected areas are polygons ≥ 5 km^2^ and ≤ 500 m elevation from the World Database on Protected Areas (UNEP-WCMC and IUCN 2023). Highland target areas are patches (≥ 5 km^2^) of upper-montane (≥ 1500 m elevation) contiguous forest with medium or high Forest Landscape Integrity Index values (Grantham et al. 2020).

**S1 Table. Protected area coverage and connectivity in 2020 (Álvarez Malvido et al. 2021)**

| **Country** | **Protected (%)** | **Protected + connected (%)** |
| --- | --- | --- |
| Belize | 36 | 27 |
| Colombia | 17 | 5 |
| Costa Rica | 27 | 12 |
| El Salvador | 7 | 4 |
| Guatemala | 20 | 7 |
| Honduras | 23 | 13 |
| Mexico | 14 | 3 |
| Nicaragua | 35 | 20 |
| Panama | 20 | 10 |

**S2 Table. Start and end nodes by country**

| **Country** | **Start nodes housed** | **End nodes housed** |
| --- | --- | --- |
| Belize | 49 | 0 |
| Colombia^ | 61 | 151 |
| Costa Rica | 56 | 20 |
| El Salvador | 14 | 6 |
| Guatemala | 56 | 183 |
| Honduras | 31 | 52 |
| Mexico^ | 110 | 105 |
| Nicaragua | 46 | 2 |
| Panama | 52 | 10 |

^ Values are not for the entire country; our study area only included portions of southeastern Mexico and northwestern Colombia

**S3 Table. Conductance values by land cover class**

| **Land cover class** | **Conductance** |
| --- | --- |
| Forest | 1000* |
| Shrubland | 150 |
| Grassland/pasture | 30 |
| Cropland | 30 |
| Bare/sparse vegetation | 40 |
| Herbaceous wetland | 20 |
| Mangrove | 500 |
| Developed | NA |
| Snow/ice | NA |
| No data | NA |

* multiplied by forest biomass modifier

**S4 Table. Ecological characteristics and conservation status variables used to compare potential climate adaptation corridors**

| **Corridor variables** | **Start node variables** | **End node variables** |
| --- | --- | --- |
| Length (km) | Area (km^2^) | Area (km^2^)* |
| Elevational range (m) | Elevational range (m) | Elevational range (m) |
| Protection (%) |  | Protection (%) |
| Number of overlapping protected areas* |  |  |
| Number of overlapping KBAs |  |  |
| Mean forest biomass (Mt C) |  |  |

* not included in climate corridor priority index calculation due to variable redundancy

**References**

Álvarez Malvido M, Lázaro C, De Lamo X, Juffe-Bignoli D, Cao R, Bueno P, et al. (Editores). 2021. Latin America and the Caribbean Protected Planet Report 2020. Mexico City, Mexico; Cambridge UK; Gland, Switzerland; Bogota, Colombia: Red-Parques, UNEP-WCMC, WCPA-IUCN, WWF, CONANP and IAPA Project.

Grantham HS, Duncan A, Evans TD, Jones KR, Beyer HL, Schuster R, et al. Anthropogenic modification of forests means only 40% of remaining forests have high ecosystem integrity. Nature communications. 2020 Dec 8;11(1):5978.

UNEP-WCMC and IUCN. 2023. Protected Planet: The World Database on Protected Areas (WDPA), June 2023, Cambridge, UK: UNEP-WCMC and IUCN. Available at: [www.protectedplanet.net](http://www.protectedplanet.net).
